# Effects of nordic walking training on gait and exercise tolerance in male ischemic heart disease patients

**DOI:** 10.1038/s41598-024-62109-9

**Published:** 2024-05-16

**Authors:** Agnieszka Szpala, Sławomir Winiarski, Małgorzata Kołodziej, Ryszard Jasiński, Andrzej Lejczak, Dariusz Kałka, Karolina Lorek, Jacek Bałchanowski, Sławomir Wudarczyk, Marek Woźniewski, Bogdan Pietraszewski

**Affiliations:** 1 Department of Biomechanics, Wroclaw University of Health and Sport Sciences, Mickiewicza 58 Street, 51-684, Wrocław, Poland; 2 Department of Human Biology, Wroclaw University of Health and Sport Sciences, Paderewskiego 35 Avenue, 51-612 Wrocław, Poland; 3 Department of Physiotherapy in Surgical Medicine and Oncology, Wroclaw University of Health and Sport Sciences, Paderewskiego 35 Avenue, 51-612 Wrocław, Poland; 4 Department of Physiotherapy in Internal Diseases, Wroclaw University of Health and Sport Sciences, Paderewskiego 35 Avenue, 51-612 Wrocław, Poland; 5 Department of Kinesiology, Wroclaw University of Health and Sport Sciences, Paderewskiego 35 Avenue, 51-612 Wrocław, Poland; 6 Department of Fundamentals of Machine Design and Mechatronics Systems, Wroclaw University of Science and Technology, Łukasiewicza 7/9 Street, 50-371 Wrocław, Poland

**Keywords:** Gait analysis, Heart diseases, Nordic walking, Training, Symmetry, MEMS (mechatronic sensors), Cardiology, Health care

## Abstract

This technique-focused observational study explores the impact of a 6-week Nordic Walking (NW) program on physiological and biomechanical aspects in ischemic heart disease (IHD) patients. Twelve male IHD patients (66.2 ± 5.2 years, 12.2 ± 7.5 years of disease duration) were evaluated pre- and post-training for (i) gait parameters, (ii) exercise tolerance using electrocardiographic (ECG) stress test, (iii) a 6-min walk test (6MWT). The NW training, adhering to IHD patient guidelines, involved a 100-m walk at a self-selected, preferred speed without sticks, with classic NW sticks and mechatronic sticks. A mechatronic measuring system, specifically engineered for measuring, diagnosing and monitoring the patient's gait, was integrated into mechatronic sticks. Post-training, significant enhancements were observed in ECG stress test duration, metabolic equivalency, and 6MWT distance, irrespective of the stick type. However, no significant changes were noted in spatiotemporal parameters concerning the measured side, stick utilisation, or type. The results suggest that NW training boosts exercise capacity and refines gait mechanics in male IHD patients. However, the improvement in exercise capacity was not linked to changes in gait mechanics from NW training but rather to the movement during NW gait. Hence, the key to enhancing exercise capacity in IHD patients is the movement during NW gait, not the quality of gait mechanics.

## Introduction

Walking training is the basis of rehabilitation in many medical specialities. Currently, the most recommended training is Nordic Walking (NW), which engages muscles to a greater extent than free walking, causing up to a 15-fold increase in their activity^1–3^. During NW, the range of movements in the joints increases, contributing to increased speed and safety due to better body balance^4^. Knobloch and Vogt^5^ research show that the frequency of injuries was 0.9/1000 h of training, and falls occurred with a frequency of 0.2/1000 h of training. NW training results in a higher level of maximal oxygen uptake (VO_2_ max)—up to 22%, a 16% increase in heart rate (HR) and 22% higher energy expenditure compared to free walking^1,6–8^. NW training in patients with Ischemic Heart Disease (IHD) is indicated regardless of the method of its treatment, both after percutaneous transluminal coronary angioplasty (PTCA) and after coronary artery bypass graft (CABG) surgery. The training can be implemented early after acute coronary syndrome and in subsequent stages of cardiac rehabilitation. NW is a safe form of walking training, does not cause undesirable cardiovascular effects that require a reduction in training intensity or interruption^9–12^ and improves functional efficiency, especially dynamic balance and upper and lower body endurance^9,13^.

Our previous studies of a group of patients with heart failure confirmed that the NW technique increases the intensity of training even in the case of people with low exercise tolerance. The studies showed that patients with heart failure in New York Heart Association (NYHA) class-II tolerated submaximal treadmill walking using the NW technique well. The walking did not increase the symptoms of dyspnoea, myocardial ischemia or arrhythmia. All subjects completed the test without complications of the cardiovascular system, obtaining higher values of hemodynamic parameters than during free walking^14^. Our previous research on walking training in patients with intermittent claudication also shows that the correct gait pattern assessed with kinematic and spatiotemporal parameters significantly impacts rehabilitation effectiveness^15–17^.

Taking into account the biomechanical parameters in the walking training of patients with cardiovascular diseases is vital as these patients are diagnosed with walking disorders. This applies primarily to the stabilisation of the knee joint and rebound, showing a relationship with decreased exercise tolerance in patients with cardiovascular diseases. This is particularly important in the case of the functioning of the ankle joint, where approximately 60% of the "propelling force" during gait is generated. Weakness of the calf muscles causes dysfunction of this joint and, thus, a decrease in the "propelling force" of gait, which results, among others, in a decrease in speed^18,19^. Gait speed, stride length, gait cycle and step length are also lower in these patients compared to healthy individuals^20,21^. However, in previous studies, the authors did not refer to the NW gait technique, nor did they precisely describe at what level, according to the International Nordic Walking Federation (INWA) guidelines, the training programs were conducted based on kinematic and spatiotemporal parameters. They only provided their physiological characteristics. This resulted in the difficulty of analysing these parameters in real-time using classic NW sticks. In this case, mechatronic sticks equipped with measurement and signalling systems that enable real-time measurement and feedback to the user about the walking technique may be helpful.

Therefore, the study aims to assess the relationship between the physiological and biomechanical effects of NW training in patients with IHD. It was assumed that the physiological results of this training would be related to the gait technique.

## Material and methods

### Research material

The study adopted a convenience sampling approach, focusing on the qualitative aspects of the changes in gait technique and exercise tolerance. The study was conducted in 12 men aged 57 to 71 (mean 66.2 years) suffering from ischemic heart disease (IHD) who underwent a 6-week NW training. All the patients were on beta-blocker medication. The exclusion criteria centred around any conditions that precluded the possibility of engaging in walking training. A weak differentiation characterised the study group regarding age, height, and body weight, as indicated by the calculated values of the coefficient of variation—7.85%, 3.88%, and 12.47%, respectively. This shows an excellent homogeneity of the group. This coefficient was 61.47% only for the disease's duration, proving a moderate differentiation of the variable. The disease duration ranged from 31 to 2 years, with an average of 12 years. Nine patients underwent PTCA, two CABG, and one single CABG (Table 1). None of the patients had symptoms of heart failure. All the participants had a similar level of NW gait skills.

**Table 1:** Characteristics of the study group.

Case	Age (years)	Body height (m)	Body weight (kg)	Disease duration (years)	PTCA/CABG
1	64	1.82	104	31	–
2	66	1.74	74	12	+/+
3	74	1.84	81	17	+/−
4	68	1.80	98	2	+/−
5	69	1.67	77	7	+/−
6	57	1.75	79	9	+/−
7	69	1.65	89	9	−/+
8	57	1.71	78	5	+/−
9	71	1.61	78	8	+/−
10	67	1.76	88	11	+/−
11	61	1.79	108	14	+/+
12	71	1.71	93	21	−/−
Mean ± SD	66.2 ± 5.2	1.738 ± .067	87.3 ± 10.9	12.2 ± 7.5	Total: 9/3

*SD* standard deviation, *PTCA* percutaneous transluminal coronary angioplasty, *CABG* coronary artery bypass grafting.

**Figure 1:**
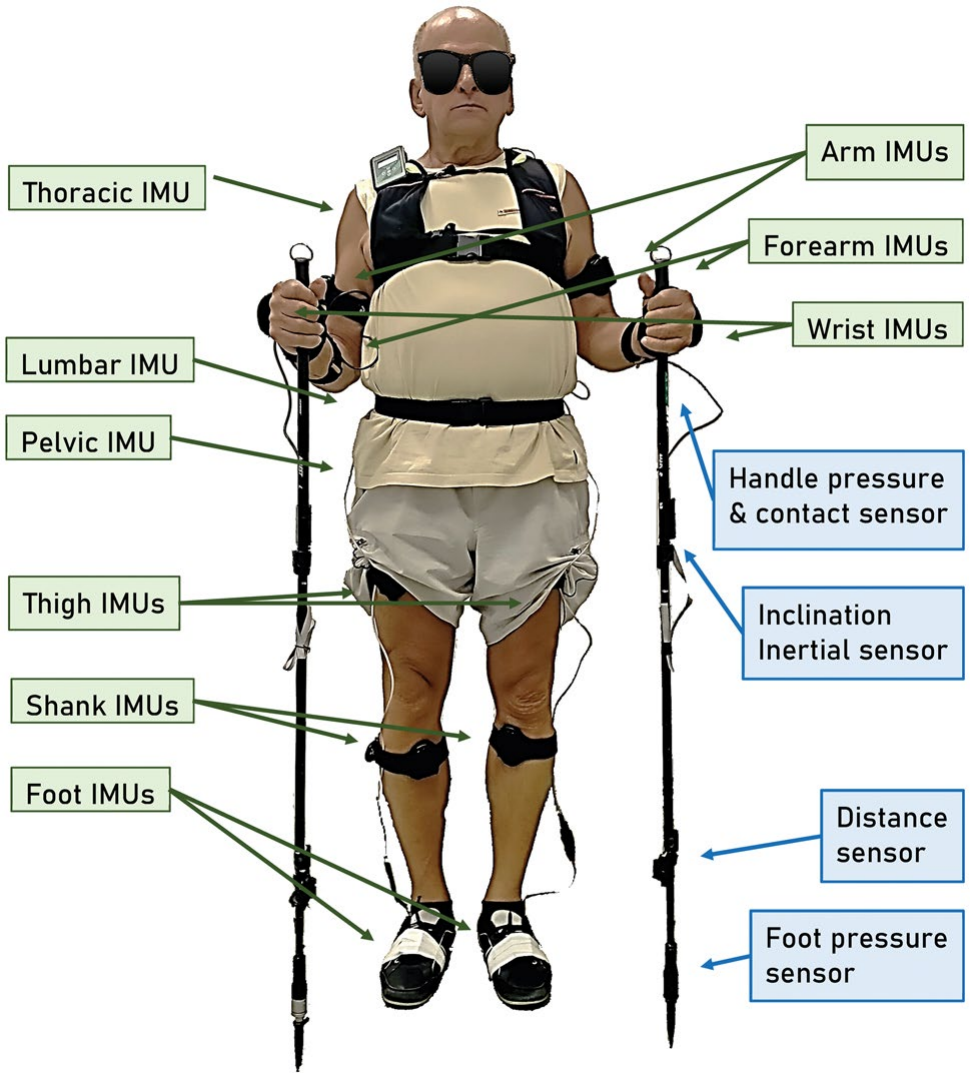
Illustration of sensors placement on the body and the NW poles. Three inertial motion sensors (IMUs) were placed for each of the upper and lower extremities (right/left) and 3 IMUs in the area of the spine (on the spinous process of the 7th cervical vertebra and 7th thoracic vertebra and in the centre of the sacrum).

The participants were informed about the aims and methodology used in the experiment and gave written informed consent for participation in the investigation. Informed consent was obtained from the participant in Fig. 1 to publish his image in an open-access publication. The Senate Committee on Research Ethics of the Wroclaw University of Health and Sport Sciences approved the experiment (Reg.No:40/2019) as conducted following the Declaration of Helsinki.

### Testing method

This study adopted a technique-focused observational approach, chosen for its ability to provide in-depth insights into the biomechanics of walking and its immediate physiological effects. The research design was observational in nature, with a focus on qualitative analysis. All patients were measured before and after training using the MyoMotion analysis system (Noraxon Inc., USA). During the measurement, inertial motion units (IMU) were placed on the tested person's body (Fig. 1). IMU sensors enabled data recording and comprehensive analysis with a sampling frequency of 200 Hz^22^.

The MyoMotion system, like any inertial measurement unit IMU-based system, has intrinsic limitations when compared to optoelectronic or video-based methods^23,24^. IMU systems can be susceptible to drift over time and may be affected by magnetic disturbances in the environment^24^. Moreover, while IMUs provide valuable data on segmental motion through space, they lack the spatial resolution of optoelectronic systems, which can more precisely measure the position of markers in a laboratory setting^23,25^. To minimise these potential biases, we employed several strategies: (1) Before initiating the study, the MyoMotion system was calibrated according to the manufacturer's guidelines, and a validation study was conducted to compare its output with an optoelectronic system for a subset of common gait parameters^26,27^. (2) We applied data filtering and processing techniques to mitigate the impact of signal noise and drift. This included the use of system-build quaternion-based algorithms for orientation estimation, which are known to reduce the cumulative error in angular measurements. (3) All participants were assessed under identical conditions, and the same trained personnel conducted all measurements to ensure consistency. This approach helped reduce the variability that could arise from procedural differences.

The subject's task was to cover a 100-m distance with three types of walking—walking without sticks, walking with classic NW sticks, and walking with mechatronic sticks. The basic step with NW sticks took place after a short instruction given by an NW instructor. The walk took place on an artificial turf pitch with a preferred speed. Two passes were made for each gait, which allowed for an average of 70 complete gait cycles per pass.

The construction of mechatronic sticks has been thoroughly described in our previous studies^28,29^. For the purpose of this study, it is essential to understand that these are not ordinary Nordic Walking poles but are enhanced with sophisticated technology aimed at precise gait analysis. Specifically, each classic Nordic Walking pole is equipped with two nine-axis inertial sensors, which include a three-axis gyroscope, accelerometer, and magnetometer. These sensors are crucial for capturing detailed movements and orientations of the pole during walking. Additionally, the foot of the pole is fitted with a pressure sensor capable of measuring the force exerted along the pole's axis, providing insight into the ground reaction forces during the pole's contact with the surface. The handle of the pole incorporates a contact sensor to detect grip dynamics. Furthermore, to enrich the gait data, each pole is also furnished with distance sensors, comprising two optical and two ultrasonic sensors, positioned at both the foot and handle of the pole. This arrangement allows for an unparalleled level of detail in recording the spatial and temporal aspects of gait, thereby facilitating a comprehensive analysis of the walking pattern. Signal tests from the inertial, pressure, contact, and distance sensors also confirmed sufficient accuracy^28,30^. The difference in mass between classic and mechatronic sticks was 100 g, without changing the position of the center of gravity. It was possible to adjust the length of the mechatronic stick, which was adapted to the participant's body height, so that in a standing position, the person holding the stick perpendicular to the ground had the elbow joint bent at an angle of 90 degrees and the forearm parallel to the ground^31^.

The following parameters were recorded separately for the right/left (RT/LT) lower/upper limb:

1. spatiotemporal parameters related to the step cycle (in %): stance phase duration, loading response duration, single support duration, pre-swing duration, swing phase duration, and double stance duration; step time (in ms), stride time (ms), step length (cm), stride length (cm), velocity (m/s) and step frequency (cadence; in steps/min) were also recorded;
2. range of motion (ROM) expressed in angular degrees at the following joints: shoulder (flexion–extension, abduction–adduction, internal–external rotation), elbow (flexion–extension), wrist (flexion–extension, radial-ulnar, supination-pronation), hip (flexion–extension, abduction–adduction, internal–external rotation), knee (flexion–extension), ankle (dorsiflexion-plantarflexion, inversion-eversion, abduction–adduction).

An electrocardiographic (ECG) stress test was performed on a treadmill while monitoring a patient's condition. Blood pressure and HR were measured and recorded at least every 2–3 min. The end of the test occurred after reaching the assumed HR or meeting the cardiac criteria for stopping the test (Table 2)^32^.

**Table 2:** A protocol according to Bruce's modification.

Degree	Phase time (min)	Speed (km/h)	Treadmill incline (%)	MET
1	3	2.7	0	2.3
2	3	2.7	5	3.5
3	3	2.7	10	4.6
4	3	4.0	12	7.1
5	3	5.5	14	10.2
6	3	6.8	16	13.5

*MET* metabolic equivalent of task.

Another measured parameter was exercise tolerance. It was measured using an electrocardiographic Modified Bruce Protocol and a 6-Minute Walk Test (6MWT)^33,34^. The 6MWT was performed over a designated distance of 30 m without the use of poles, in line with the standard protocol for evaluating exercise tolerance to submaximal effort. The subject walked back and forth (shuttle walk) for 6 min at a pace of their choice, as fast as possible, but without running^35^.

### NW training

The training protocol was designed according to the current guidelines for patients with IHD^36^. It involved endurance training conducted in an interval system, which took place 3 times a week for one hour over 6 weeks. The recording of the tested parameters took place before the start of the NW training (Test 1) and after its completion (Test 2). Throughout the program, each patient underwent 18 one-hour training sessions. Training loads were selected individually for each person based on the result of the ECG exercise test, 6MWT, hemodynamic response to physical effort and subjective feelings of the patient on the simplified Borg RPE Scale^37^ (6 to 20, where 6 indicates no exertion and 20 represents maximal exertion). The 6-week rehabilitation program was divided into 2 equal stages of 3 weeks each. The detailed training program is presented in Table 3. It was carried out in controlled conditions under the supervision of a physiotherapist (an NW instructor) with a gradually increasing load. The patients were equipped with NW sticks (Campra with a clip, KV+, Switzerland) individually adjusted to the body height of each user.

**Table 3:** NW training protocol.

Training stage	I basic	II improvement
Stage duration	3 weeks	3 weeks
Duration of training unit	60 min	60 min
Parts of a training unit and their duration	Warm-up (W)—10 min Interval (I)—6 min Active break (AB)—6 min Final part (FP)—8 min	
Number AB	3	3
Number I	4	4
Distance I [m]	500	600
Intensity I	60–80% HRR	75–80% HRR
Program I	walking with poles using basic technique	improving walking technique
Intensity W	50–60% HRR	60–70% HRR
Program W	Breathing exercises with poles Fitness exercises Active dynamic exercises with poles	
Intensity AB	40–50% HRR	40–50% HRR
Program AB	Breathing and relaxation exercises in a standing and/or sitting position (depending on the degree of fatigue) Coordination and balance exercises using poles	
Intensity FP	55% HRR	55% HRR
Program FP	Breathing exercises with poles Relaxing exercises	

*HRR* heart rate reserve.

Regarding the control of training intensity, it was individually tailored for each participant based on the results from the ECG stress test using the modified Bruce protocol. The target training heart rate was set at 60–85% of HR reserve, consistent with established practices for cardiovascular training adaptations that ensure both safety and efficacy. During rest intervals between exercise sessions, heart rate was closely monitored to remain within the prescribed range. The maximum distance achieved in the 6MWT test was used to calibrate each individual’s capacity for sustained aerobic exercise, thereby aiding in the customisation of the exercise load. The training loads were expressed as a percentage of the maximal distance covered in the 6MWT, where practical, to further personalise the training intensity. Additionally, we utilised ratings of perceived exertion (RPE) and hemodynamic responses as supplementary measures to fine-tune the exercise prescriptions throughout the training period.

### Statistical analysis

The variables' normal distribution was checked using the Shapiro–Wilk test. Differences between the results of functional efficiency measurements before and after the intervention were verified with the paired sample t-test or the Wilcoxon test in the absence of normal distribution. Changes in gait parameters between the pre-post tests were analysed using the analysis of variance for repeated measurements. The analysis considered the type of gait and the side of the measurement as possible factors differentiating the examined variables and their changes. The homogeneity of variance was verified by the M-Box test. Tukey's post hoc test was used for multiple comparisons. Friedman's and Dunn Bonferroni's post hoc tests were used in case of violation of ANOVA assumptions. Partial eta squared (η_p_^2^) was used to assess the effect size of the ANOVA results following the recommendations of Richardson^38^. Cohen's d was calculated for paired observations and standardised for the correlation strength between the measurements^39^. For Cohen's d = 1.2, the test's estimated power was 0.80. Relationships between changes in gait parameters and changes in the results of fitness tests were assessed with Spearman's ρ. All the analyses were performed using Statistica 13.3.0 (TIBCO Software Inc.). The statistical significance of the results was accepted at p < 0.05.

## Results

### Physiological parameters

The results of all measurements in tests before and after the 6-week NW training are presented in Table 4. After the 6-week NW training programme, there was a notable extension in the duration of the ECG stress test and an increase in the metabolic equivalent. Furthermore, a significant improvement was observed in the distance covered during the 6-min walk test (6MWT).

**Table 4:** Results of the ECG test and the 6MWT distance of cardiac patients before (Test 1) and after the 6-week NW training (Test 2) (n = 12).

Parameters	Test 1 M ± SD	Test 2 M ± SD	p-value (t-test/*Wilcoxon test*)	Cohen's d
Time, (s)	886.92 ± 108.74	960.17 ± 99.24	< *0.001*	12.18
MET	10.57 ± 2.05	11.63 ± 2.14	0.018	1.91
6MWT, (m)	499.25 ± 52.20	570.25 ± 53.80	< *0.001*	2.73

*MET* metabolic equivalent of task, *6MWT* a 6-min walk test, *M* mean, *SD* standard deviation.

Italics indicate the results of non-parametric tests;

In addition, the NW training Borg scale results presented a notable distribution among the participants' perceived exertion levels. Out of the 12 participants, a significant majority, specifically 9 participants, reported their exertion levels to be between 14 and 15 on the simplified Borg scale. On the other hand, 3 participants recorded scores between 16 and 17.

### Kinematic parameters

In the case of kinematic parameters (Table 5), in the second test, higher values were observed than in the first one for ankle dorsi-/plantarflexion and wrist supination-pronation during free walking for both sides of the measurement, ankle inversion-eversion for the left side of the measurement both with and without both types of sticks, hip flexion–extension and shoulder internal–external rotation regardless of measurement side and poles used, hip abduction–adduction and hip internal–external rotation for the left side only in normal walking, wrist flexion–extension for the left side in free walking and with standard sticks and shoulder flexion–extension for the right side with mechatronic sticks. Elbow flexion–extension in the second measurement compared to the first one was larger in the standard gait and lower in the right limb when walking with sticks. The largest differences between the 1st and 2nd tests concerned the parameters measured for the left side during free walking. The side of measurement was a significant effect determining differences only for ankle inversion–eversion (p < 0.001), while the use of sticks or their lack differentiated changes in ankle dorsi-/plantarflexion (p < 0.001), elbow flexion–extension (p < 0.001), hip abduction–adduction (p = 0.007), hip internal–external rotation (p = 0.002), shoulder internal–external rotation (p = 0.001), and wrist supination-pronation (p < 0.001), indicating more significant differences when walking without sticks than with sticks. The types of sticks did not determine the observed changes in angular ranges.

**Table 5:** Measurement results of kinematic parameters of gait depending on its type in cardiac patients before (Test 1) and after (Test 2) the 6-week NW training (n = 12).

ROM (deg)	Type of gait	Test 1 M ± SD	Test 2 M ± SD	p-value (ANOVA/*Friedman test*)	p-value (post-hoc test)	Cohen's d
Ankle abduction–adduction LT	Free walk	13.86 ± 4.71	15.69 ± 6.39			
	Standard	21.1 ± 9.79	13.8 ± 4.08			
	Mechatron	21.92 ± 10.58	15.45 ± 5.12	*0.267*		
Ankle abduction–adduction RT	Free walk	15.89 ± 7.46	21.98 ± 10.26			
	Standard	22.95 ± 10.58	21.9 ± 8.56			
	Mechatron	24.36 ± 13.64	20.44 ± 8.91			
Ankle dorsiflexion–plantarflexion LT	Free walk	29.2 ± 9.03	51.14 ± 11.58		< 0.001	1.81
	Standard	46.68 ± 14.76	51.79 ± 14.55		ns	
	Mechatron	44.58 ± 15.14	51.22 ± 13.48	< 0.001	ns	
Ankle dorsiflexion–plantarflexion RT	Free walk	27.87 ± 7.39	51.86 ± 8.48	η_p_^2^ = 0.44	< 0.001	3.17
	Standard	46.81 ± 13.98	58.73 ± 15.64		ns	
	Mechatron	45.45 ± 11.17	50.45 ± 13.26		ns	
Ankle inversion–eversion LT	Free walk	11.16 ± 4.36	17.72 ± 4.43		0.023	1.23
	Standard	15.28 ± 5.82	20.76 ± 4.97		0.025	1.21
	Mechatron	17.97 ± 6.91	25.21 ± 3.17	< 0.001	0.007	1.22
Ankle inversion–eversion RT	Free walk	15.65 ± 4.64	19.88 ± 6.95	η_p_^2^ = 0.24	ns	
	Standard	21.74 ± 7.24	21.84 ± 7.13		ns	
	Mechatron	22.7 ± 6.76	18.9 ± 6.62		ns	
Elbow flexion–extension LT	Free walk	29.97 ± 11.83	39.91 ± 14.3		*0.028*	1.27
	Standard	57.7 ± 21.76	41.38 ± 14.93		*ns*	
	Mechatron	59.91 ± 21.24	43.01 ± 18.28	< *0.001*	*ns*	
Elbow flexion–extension RT	Free walk	22.26 ± 11.49	30.46 ± 10.25		0.041	0.66
	Standard	54.53 ± 23.4	36.48 ± 7.71		0.004	1.22
	Mechatron	54.87 ± 20.91	37.38 ± 10.01		0.016	1.22
Hip abduction–adduction LT	Free walk	14.7 ± 4.73	22.55 ± 4.67		0.027	1.18
	Standard	21.1 ± 3.88	23.32 ± 6.28		ns	
	Mechatron	23.13 ± 7.44	23.49 ± 6.3	0.021	ns	
Hip abduction–aduction RT	Free walk	15.43 ± 3.92	19.66 ± 4.31	η_p_^2^ = 0.08	ns	
	Standard	20.84 ± 5.65	20.9 ± 3.24		ns	
	Mechatron	24.04 ± 6.04	21.91 ± 3.63		ns	
Hip flexion–extension LT	Free walk	49.56 ± 6.82	66.53 ± 8.79		< 0.001	3.41
	Standard	60.19 ± 8.7	72.05 ± 8.93		0.003	3.88
	Mechatron	59.63 ± 10.25	72.97 ± 9.64	< 0.001	< 0.001	1.90
Hip flexion–extension RT	Free walk	51.04 ± 9.3	70.39 ± 12.09	η_p_^2^ = 0.73	< 0.001	2.84
	Standard	62.61 ± 8.71	75.13 ± 12.16		0.001	1.97
	Mechatron	61.17 ± 9.7	75.95 ± 12.02		< 0.001	1.72
Hip internal–external rotation LT	Free walk	18.82 ± 5.74	26.63 ± 9.26		0.032	0.94
	Standard	28.37 ± 8.25	28.5 ± 8.44		ns	
	Mechatron	26.88 ± 7.99	31.09 ± 8.52	0.005	ns	
Hip internal–external rotation RT	Free walk	18.42 ± 5.28	23.52 ± 7.63	η_p_^2^ = 0.12	ns	
	Standard	28.52 ± 6.13	25.01 ± 7.33		ns	
	Mechatron	24.31 ± 7.06	26.4 ± 8.74		ns	
Knee flexion–extension LT	Free walk	68.8 ± 4.71	72.69 ± 7.17			
	Standard	69.16 ± 6.67	69.96 ± 6.62			
	Mechatron	68.72 ± 7.77	70.08 ± 6.7	0.069		
Knee flexion–extension RT	Free walk	66.98 ± 7.68	70.79 ± 5.73			
	Standard	68.42 ± 6.5	69.54 ± 7.37			
	Mechatron	67.17 ± 6.44	67.56 ± 8.7			
Shoulder abduction–adduction LT	Free walk	9.08 ± 7.88	9.61 ± 5.96			
	Standard	12.35 ± 9.88	11.22 ± 7.51			
	Mechatron	13.77 ± 8.76	10.19 ± 4.25	*0.524*		
Shoulder abduction–adduction RT	Free walk	8.24 ± 3.83	7.65 ± 3.12			
	Standard	12.74 ± 4.5	18.78 ± 21.67			
	Mechatron	10.76 ± 4.89	15.59 ± 15.16			
Shoulder internal–external rotation LT	Free walk	21.47 ± 6.41	83.37 ± 16.06		< 0.001	3.68
	Standard	47.83 ± 24.68	83.04 ± 24.16		0.006	1.17
	Mechatron	46.11 ± 24.18	74.35 ± 14.93	< 0.001	0.032	1.06
Shoulder internal–external rotation RT	Free walk	19.53 ± 9.09	83.6 ± 20.4	η_p_^2^ = 0.71	< 0.001	4.58
	Standard	50.16 ± 17.33	89.14 ± 19.26		0.001	1.91
	Mechatron	49.93 ± 16.54	86.64 ± 13.36		0.003	2.06
Shoulder flexion–extension LT	Free walk	15.28 ± 8.25	17.99 ± 7.87		*ns*	
	Standard	17.29 ± 6.3	21.35 ± 8.97		*ns*	
	Mechatron	16.35 ± 5.77	23.25 ± 9.34	*0.006*	*ns*	
Shoulder flexion–extension RT	Free walk	12.66 ± 6.23	17.09 ± 7.38		*ns*	
	Standard	17.01 ± 7.1	28.49 ± 15.54		*ns*	
	Mechatron	16.11 ± 7.25	27.65 ± 13.44		*0.041*	0.84
Wrist flexion–extension LT	Free walk	8.39 ± 5.23	13.61 ± 12.07		*0.028*	0.40
	Standard	12.19 ± 6.36	17.13 ± 7.97		*0.026*	1.07
	Mechatron	12.99 ± 6.04	15.8 ± 6.63	*0.017*	*ns*	
Wrist flexion–extension RT	Free walk	6.74 ± 3.91	8.03 ± 5.04		*ns*	
	Standard	10.28 ± 5.47	15.21 ± 8.19		*ns*	
	Mechatron	9.86 ± 4.2	10.82 ± 4.53		*ns*	
Wrist radial–ulnar LT	Free walk	8.79 ± 3.94	16.25 ± 6.9			
	Standard	24.52 ± 8.43	23.89 ± 9.59			
	Mechatron	24.53 ± 13.19	25.8 ± 12.35	*0.149*		
Wrist radial–ulnar RT	Free walk	7.05 ± 4.54	15.73 ± 8.54			
	Standard	21.03 ± 9.52	22.31 ± 8.44			
	Mechatron	24.1 ± 13.09	20.23 ± 4.93			
Wrist supination–pronation LT	Free walk	13.56 ± 7.69	72.84 ± 15.38		*0.002*	4.16
	Standard	70.05 ± 38.75	89.02 ± 10.64		*ns*	
	Mechatron	60.8 ± 44.26	90.93 ± 9.52	< *0.001*	*ns*	
Wrist supination–pronation RT	Free walk	8.49 ± 5.78	79.85 ± 18.93		*0.002*	4.28
	Standard	64.06 ± 40.97	90.49 ± 11.11		*ns*	
	Mechatron	63.05 ± 43.34	89.4 ± 7.96		*ns*	

Italics indicate the results of non-parametric tests.

*ROM* range of motion, *M* mean, *SD* standard deviation, *LT, RT* left and right sides, respectively, *η*_*p*_^*2*^ partial eta squared for the effect of the repeated measurement (ANOVA), *ns* no statistical significance at p < 0.05.

### Spatiotemporal parameters

Changes in spatiotemporal parameters in the second measurement compared to the first were noted only in the case of walking with regular and mechatronic sticks. Measurement results of kinematic parameters of gait depending on its type in cardiac patients before (Test 1) and after (Test 2) the 6-week NW training (n = 12) are presented in Table 6. Changes in spatiotemporal parameters in the second measurement compared to the first were noted only in the case of walking with regular and mechatronic sticks.

**Table 6:** Measurement results of spatiotemporal gait parameters depending on its type in cardiac patients before (Test 1) and after (Test 2) the 6-week NW training (n = 12).

	Type of gait	Test 1 M ± SD	Test 2 M ± SD	p-value (ANOVA*/Friedman test*)	p-value (post-hoc test)	Cohen's d
Cadence, step/min	Free walk	114.39 ± 4.3	114.07 ± 6.43			
	Standard	110.84 ± 8.87	111.75 ± 6.26	0.625		
	Mechatron	108.6 ± 7.7	110.31 ± 5.13			
Double stance, %	Free walk	19.61 ± 2.78	18.92 ± 3.01		ns	
	Standard	18.83 ± 2.64	16.67 ± 2.56	0.001	0.015	1.73
	Mechatron	19.07 ± 2.86	16.84 ± 2.09	η_p_^2^ = 0.27	0.013	0.75
Load response LT, %	Free walk	9.93 ± 1.51	9.54 ± 1.44		ns	
	Standard	9.55 ± 1.85	8.48 ± 1.63		0.043	0.64
	Mechatron	9.45 ± 1.56	8.31 ± 1.19	< 0.001	0.032	0.58
Load response RT, %	Free walk	9.66 ± 1.98	9.35 ± 1.72	η_p_^2^ = 0.20	ns	
	Standard	9.18 ± 1.57	8.08 ± 1.42		0.038	1.63
	Mechatron	9.59 ± 1.64	8.46 ± 1.25		0.032	0.90
Pre-swing LT, %	Free walk	9.69 ± 1.99	9.22 ± 1.64		*ns*	
	Standard	9.7 ± 1.8	8.24 ± 1.54		*0.002*	3.98
	Mechatron	9.61 ± 1.66	8.48 ± 1.25	< *0.001*	*ns*	
Pre-swing RT, %	Free walk	9.94 ± 1.5	9.72 ± 1.76		*ns*	
	Standard	9.21 ± 1.52	8.55 ± 1.51		*ns*	
	Mechatron	9.48 ± 1.55	8.45 ± 1.1		*ns*	
Single support LT, %	Free walk	39.57 ± 1.94	40.11 ± 1.35		*ns*	
	Standard	39.13 ± 2.71	41.04 ± 1.71		*0.034*	1.00
	Mechatron	39.59 ± 1.75	41.18 ± 1.41	*0.005*	*0.019*	0.95
Single support RT, %	Free walk	40.85 ± 1.44	40.82 ± 2.25		*ns*	
	Standard	41.62 ± 1.66	42.02 ± 1.32		*ns*	
	Mechatron	41.34 ± 1.61	41.88 ± 1.04		*ns*	
Stance phase LT, %	Free walk	59.21 ± 1.46	58.92 ± 1.85		*ns*	
	Standard	58.43 ± 1.61	57.76 ± 1.25		*0.023*	1.34
	Mechatron	58.67 ± 1.68	58 ± 1.17	*0.001*	*ns*	
Stance phase RT, %	Free walk	60.46 ± 1.92	59.89 ± 1.34		*ns*	
	Standard	60.03 ± 1.7	58.66 ± 1.81		*0.041*	0.95
	Mechatron	60.43 ± 1.74	58.8 ± 1.45		*0.019*	0.95
Step length LT, cm	Free walk	72.1 ± 7.43	76.63 ± 8.69			
	Standard	78.69 ± 8.01	80.36 ± 27.64			
	Mechatron	78.89 ± 9.05	80.13 ± 25.45	*0.818*		
Step length RT, cm	Free walk	76.02 ± 10.21	79.81 ± 9.22			
	Standard	82.32 ± 8.53	74.59 ± 26.95			
	Mechatron	79 ± 8.37	79.51 ± 29.02			
Step time LT, ms	Free walk	531.1 ± 25.8	530.5 ± 33.1			
	Standard	557.1 ± 44.6	544.2 ± 35.7			
	Mechatron	566.7 ± 47.78	549.8 ± 27.6	0.418		
Step time RT, ms	Free walk	520.1 ± 23.2	526.1 ± 35.2			
	Standard	535.9 ± 40.9	534.9 ± 31.9			
	Mechatron	544.5 ± 34.01	541.67 ± 28.3			
Stride length, cm	Free walk	172.3 ± 49.63	187.26 ± 57.3			
	Standard	185.9 ± 56.7	172.9 ± 64.88	*0.883*		
	Mechatron	170.3 ± 42.0	162.7 ± 56.4			
Stride time, ms	Free walk	1051.2 ± 41.1	1056.6 ± 66.2			
	Standard	1093.0 ± 82.7	1079.1 ± 64.8	0.555		
	Mechatron	1111.2 ± 80.0	1091.4 ± 53.6			
Swing phase LT, %	Free walk	40.79 ± 1.46	41.08 ± 1.85		ns	
	Standard	41.57 ± 1.61	42.24 ± 1.25		ns	
	Mechatron	41.33 ± 1.68	42 ± 1.17	< 0.001	ns	
Swing phase RT, %	Free walk	39.54 ± 1.92	40.11 ± 1.34	η_p_^2^ = 0.22	ns	
	Standard	39.97 ± 1.7	41.34 ± 1.81		0.007	0.94
	Mechatron	39.57 ± 1.74	41.2 ± 1.45		0.001	0.95
Velocity, m/s	Free walk	1.65 ± 0.54	1.8 ± 0.63			
	Standard	1.74 ± 0.68	1.63 ± 0.61	*0.943*		
	Mechatron	1.56 ± 0.5	1.51 ± 0.49			

Italics indicate the results of non-parametric tests.

*M* mean, *SD* standard deviation, *LT, RT* left and right sides, respectively, *η*_*p*_^*2*^ partial eta squared for the effect of the repeated measurement (ANOVA), *ns* no statistical significance at p < 0.05.

No differences were found during the free walk. The following values have decreased: double stance, load response regardless of the measured side and used sticks, pre-swing for the left side when walking with regular sticks, stance phase when walking with mechatronic sticks only for the right side and standard sticks for both sides. Higher values in the second test than in the first were recorded only for single support LT and swing phase RT, regardless of the type of sticks. The measured side, the use of sticks or their lack and type did not significantly differentiate changes in spatiotemporal parameters in the pre-post tests.

### Changes in functional fitness and gait parameters

All changes in parameters related to biomechanical gait parameters and changes in exercise capacity parameters were independent of the patients' age (p > 0.05). For most of the observed changes in biomechanical gait parameters, with a few exceptions, no association was found with changes in exercise capacity parameters (Table 7). Increasing ECG duration as a result of repeated measurements correlated with a more remarkable change in the left side of the measurements: hip flexion–extension in walking with standard walking sticks and shoulder internal–external rotation with mechatronic walking sticks, and with a smaller change in load response RT with ordinary walking sticks. MET differences were negatively correlated with changes in hip abduction–adduction LT and hip internal–external rotation LT in free walking and positively with changes in shoulder internal–external rotation LT and shoulder flexion–extension RT while walking with mechatronic sticks and with changes in wrist supination-pronation RT when free walking. Changes in 6MWT scores were only correlated with changes in ankle dorsiflexion-plantarflexion RT during free walking.

**Table 7:** Correlation coefficients ρ—spearman between changes in "functional fitness" parameters and changes in gait parameters.

Parameters	Type of gait	ΔTime	ΔMET	Δ6MWT
ΔAnkle dorsiflexion–plantarflexion LT, (deg)	Free walk	0.574	− 0.047	0.007
ΔAnkle dorsiflexion–plantarflexion RT, (deg)	Free walk	0.193	0.163	**− 0.580**
ΔAnkle inversion–eversion LT, (deg)	Free walk	− 0.221	− 0.505	− 0.455
	Standard	0.042	− 0.407	− 0.287
	Mechatron	0.088	0.051	0.182
ΔElbow flexion–extension LT, (deg)	Free walk	0.378	0.472	0.245
	Standard	− 0.392	− 0.316	− 0.182
	Mechatron	− 0.445	− 0.265	− 0.287
ΔElbow flexion–extension RT, (deg)	Free walk	− 0.060	0.105	0.091
	Standard	− 0.396	− 0.309	− 0.245
	Mechatron	− 0.308	− 0.465	− 0.168
ΔHip abduction–adduction LT, (deg)	Free walk	− 0.263	**− 0.588**	− 0.028
ΔHip flexion–extension LT, (deg)	Free walk	0.532	− 0.025	0.105
	Standard	**0.623**	0.025	− 0.063
	Mechatron	0.557	0.142	0.126
ΔHip flexion–extension RT, (deg)	Free walk	0.238	− 0.291	− 0.238
	Standard	0.308	0.036	0.077
	Mechatron	0.522	0.236	0.147
ΔHip internal–external rotation LT, (deg)	Free walk	− 0.277	**− 0.687**	− 0.399
ΔShoulder internal–external rotation LT, (deg)	Free walk	0.238	− 0.127	− 0.021
	Standard	0.560	0.469	− 0.133
	Mechatron	**0.809**	**0.618**	0.168
ΔShoulder internal–external rotation RT, (deg)	Free walk	0.305	0.163	− 0.210
	Standard	0.504	0.469	− 0.175
	Mechatron	0.427	0.276	− 0.084
ΔShoulder flexion–extension RT, (deg)	Mechatron	0.508	**0.846**	0.399
ΔWrist flexion–extension LT, (deg)	Free walk	0.060	0.338	0.413
	Standard	0.207	0.461	0.196
ΔWrist supination–pronation LT, (deg)	Free walk	− 0.179	0.047	− 0.063
ΔWrist supination–pronation RT, (deg)	Free walk	0.123	**0.588**	0.182
ΔDouble stance, (%)	Standard	− 0.109	− 0.392	0.077
	Mechatron	0.025	− 0.131	− 0.035
ΔLoad response LT, (%)	Standard	0.151	− 0.400	0.084
	Mechatron	0.004	− 0.276	0.168
ΔLoad response RT, (%)	Standard	**− 0.595**	− 0.185	− 0.406
	Mechatron	0.025	0.025	− 0.035
ΔPre− swing LT, (%)	Standard	− 0.305	− 0.309	− 0.238
ΔSingle support LT, (%)	Standard	− 0.060	0.331	− 0.112
	Mechatron	0.200	0.363	0.021
ΔStance phase LT, (%)	Standard	− 0.284	− 0.214	− 0.210
ΔStance phase RT, (%)	Standard	− 0.270	− 0.465	− 0.133
	Mechatron	− 0.053	− 0.385	− 0.028
ΔSwing phase RT, (%)	Standard	0.270	0.465	0.133
	Mechatron	0.053	0.385	0.028

*Δ* the difference in parameter values between test 1 and test 2, *LT, RT* left and right sides, respectively, *MET* metabolic equivalent, *6MWT* a 6-min walk test.

Significant correlations are marked in bold at p < 0.05.

## Discussion

The 6-week NW training significantly increased the exercise capacity of the examined patients with IHD, extending the duration of the ECG exercise test by about 8% (min. = 4%, max. = 17%), and the distance in 6MWT by about 14% (min. = 1%, max. = 32%), which resulted in an increase in MET by about 9% (min. = 0%, max. = 33%). These are lower values of increases in these parameters than those obtained by other authors^9,13^. Still, the patients had relatively high baseline values. The average MET value before the start of the NW training qualified them for the group of high efficiency, and the result obtained in 6MWT was within the range for healthy people. Typically, higher initial values ​​of the tested parameters result in lower increases due to physical training. Nevertheless, the tested parameters characterising exercise capacity showed significant increases. These variations could be attributed to several factors including individual health conditions, the level of adherence to the training protocols, and participants' baseline fitness levels. By analysing these factors, we aimed to provide insights into the potential range of outcomes from NW training and further personalise future training protocols. The use of the 6MWT, a submaximal exercise test, in the following study was instrumental in assessing the exercise tolerance relevant to everyday physical activities, devoid of the influence of assistive devices like poles.

In addition, the NW training Borg scale results present a notable distribution among the participants' perceived exertion levels. A significant majority, 9 out of the 12 participants, reported their exertion levels to be between 14 and 15 on the simplified Borg scale. These scores are considered to be in the high range, indicating that these participants perceived the activity as being moderately hard. This level of exertion suggests that while the activity was challenging, it was not at the peak of their capacity. Such scores often reflect a vigorous level of effort, where the participants are likely pushing themselves but not to the point of maximum exertion. On the other hand, 3 participants recorded scores between 16 and 17. These are notably higher scores, falling closer to the upper end of the Borg scale. Such ratings imply that these participants experienced the training as very hard, nearing their maximal effort. The distinction between these two groups is significant. The larger group with scores of 14–15 implies that the majority of participants found the activity to be strenuous but not overwhelming. In contrast, the smaller group with scores of 16–17 indicates a subset of participants who were pushing towards their maximal effort levels. This variation in perceived exertion highlights the importance of individual differences in physical conditioning, mental resilience, and personal thresholds for exertion.

The 6-week NW training also resulted in a significant increase in kinematic parameters, which are essential for gait efficiency. This concerned primarily the increase in the range of motion in the ankle joint during free walking, which constitutes 60% of the "driving" force of walking, and in the hip joint, regardless of the type of gait and in the shoulder joints during walking with mechatronic sticks, which is also a factor in increasing the efficiency and safety of walking. The results of the impact of NW training on the kinematic parameters of gait obtained by us are also confirmed by studies by other authors^4^. It should also be emphasised that the use of sticks strongly affected changes in ankle dorsiflexion-plantarflexion (η_p_^2^ = 0.22, p < 0.001), which confirms that the NW gait is an essential stimulus for increasing walking efficiency.

It was also important to show the relatively most considerable differences between the first and second tests of the parameters measured for the left side in free walking. This could indicate the activation of the left half of the body, which, due to the specificity of the disease, is used less by patients with IHD. One of the symptoms of IHD is pain behind the sternum radiating to the left shoulder, which is aggravated by physical exertion. This causes a reflex, analgesic reaction consisting in less involvement of the left half of the body in various life situations, also when walking, which can lead to a permanent asymmetry. NW training in patients with IHD may prevent these reactions or restore the symmetry of the right and left half of the body during gait.

In the case of spatiotemporal parameters, significant changes were demonstrated only during the study of walking with NW poles, which indicates the legitimacy of this type of training. A substantial decrease in the double stance value and an increase in the single support and swing phase values after a 6-week NW training in patients with IHD confirms the improvement of their walking efficiency. It is consistent with the observations of other authors. However, there was no increase in stride length and shortening of cadence, which was observed in similar studies^4^.

The increase in the value of the studied physiological and biomechanical parameters due to a 6-week NW training in patients with IHD seems to be a fairly obvious result. In contrast, the central assumption of these studies was the relationship between changes in these parameters. It was assumed that increased exercise tolerance would be associated with improving gait efficiency measured by kinematic and spatiotemporal parameters. However, this assumption was not confirmed because most changes in exercise capacity parameters resulted from 6-week NW training in patients with IHD. Apart from a few exceptions, no relationship was found with changes in the biomechanical parameters of gait. It follows that the important role in increasing the exercise tolerance of these patients was the gait movement itself, not its quality. Nevertheless, some strong correlations may be the basis for further research and suggest the existence of relationships between the increase in exercise capacity and the change in some angular and spatiotemporal parameters of gait.

With the increase in the range of hip flexion–extension when walking with standard walking sticks and shoulder internal–external rotation when walking with mechatronic walking sticks, the duration of the ECG stress test increased. While the relationship between the increase in hip flexion–extension with the duration of the stress test seems quite apparent, the relationship between shoulder internal–external rotation is challenging to explain. This is because gait is performed mainly in the sagittal plane, and movements in the frontal and transverse take place to a small extent. They are not recommended because they increase energy expenditure, shortening the time of the exercise test. However, this does not apply to walking with NW sticks because a positive correlation occurred between the increase in MET and shoulder internal–external rotation. Perhaps, during walking with sticks, movements in the transverse plane in the shoulder joint resulting from the specificity of this type of gait allow for better results of stress tests. However, this would require confirmation in further studies conducted in larger populations.

In the case of spatiotemporal parameters, the only negative correlation concerned the increase in the duration of the ECG exercise test and the load response of the right limb when walking with standard walking sticks, which seems quite obvious.

While significant enhancements in physiological parameters were evident, kinematic parameters also showed improvements, particularly in ankle dorsiflexion-plantarflexion, hip flexion–extension, and shoulder internal–external rotation. It should be noted, however, that these changes in kinematic parameters did not exhibit a direct correlation with the improvement in exercise capacity, underscoring that the benefits of NW training extend beyond mere mechanical efficiency to include substantial physiological benefits for IHD patients.

### Limitations in the conducted study

This study was initially based on the rationale that comparing participants' performance with and without NW poles would isolate the NW technique's specific effects. This approach aimed to mitigate the need for a traditional control group by facilitating a within-subject comparison across different walking conditions. However, we recognise the value of including a control group, which could have clarified the distinction between the specific effects of NW and the general benefits of increased physical activity. The study design also did not fully control for potential co-interventions, such as the participants' increased motivation and engagement in daily activities, which could have influenced the outcomes. Additionally, our analysis did not account for the degree of advancement of IHD and the activity and physical fitness levels of the examined men, factors that could significantly impact the tested parameters. The necessity for long-term observations to ascertain the durability of the observed improvements further delineates the scope for comprehensive future research. Accordingly, future studies should engage a larger cohort of IHD patients, considering the severity of the disease and their activity and physical fitness levels as independent variables to provide a more nuanced understanding of NW's impact. Moreover, conducting similar research in a group of women is essential, given that sex may influence the gait technique of patients with IHD, thereby offering critical insights into the differential effects of NW across genders.

## Conclusions

In conclusion, the present study demonstrates that NW is a useful therapeutic intervention for improving the exercise capacity and gait technique of men with IHD. The results indicate that NW training significantly impacted exercise capacity, leading to an improvement in gait technique among patients with IHD. However, the findings suggest no substantial relationship between the changes in exercise capacity and alterations in gait mechanics parameters in most patients, although a few exceptions were observed. Therefore, it appears that the primary focus of NW training in increasing exercise capacity in patients with IHD is to improve the overall movement patterns during NW gait rather than solely enhancing gait mechanics quality. These results offer valuable insights for clinicians seeking to optimise rehabilitation programs for patients with IHD by integrating NW as an effective intervention for enhancing exercise capacity and gait technique.

## Data Availability

The individual datasets are available from the corresponding author on reasonable request.

## References

[1] Pellegrini B (2015). Exploring muscle activation during nordic walking: A comparison between conventional and uphill walking. PLoS ONE.

[2] Pellegrini B (2017). Mechanical energy patterns in nordic walking: Comparisons with conventional walking. Gait Posture.

[3] Pellegrini B (2018). Muscular and metabolic responses to different Nordic walking techniques, when style matters. PLoS ONE.

[4] Roy M (2020). Nordic walking influence on biomechanical parameters: A systematic review. Eur. J. Phys. Rehabil. Med..

[5] Knobloch K, Vogt P (2006). Nordic Walking Verletzungen - Der Nordic-Walking-Daumen als neue Verletzungsentität. Sportverletzung Sportschaden.

[6] Porcari JP, Hendrickson TL, Walter PR, Terry L, Walsko G (1997). The physiological responses to walking with and without power poles™ on treadmill exercise. Res. Q. Exerc. Sport.

[7] Figard-Fabre H, Fabre N, Leonardi A, Schena F (2010). Physiological and perceptual responses to Nordic walking in obese middle-aged women in comparison with the normal walk. Eur. J. Appl. Physiol..

[8] Grainer A (2017). Physiological and perceptual responses to nordic walking in a natural mountain environment. Int. J. Environ. Res. Public Health.

[9] Cugusi L (2017). Nordic walking for individuals with cardiovascular disease: A systematic review and meta-analysis of randomized controlled trials. Eur. J. Prev. Cardiol..

[10] Piotrowicz E (2015). Home-based telemonitored Nordic walking training is well accepted, safe, effective and has high adherence among heart failure patients, including those with cardiovascular implantable electronic devices: A randomised controlled study. Eur. J. Prev. Cardiol..

[11] Rybicki JR (2015). Oxygen uptake during Nordic walking training in patients rehabilitated after coronary events. Kardiol. Pol..

[12] Vehí C (2016). Nordic walking for cardiovascular prevention in patients with ischaemic heart disease or metabolic syndrome. Medicina Clínica (English Edition).

[13] Girold S, Rousseau J, Le Gal M, Coudeyre E, Le Henaff J (2017). Nordic walking versus walking without poles for rehabilitation with cardiovascular disease: Randomized controlled trial. Ann. Phys. Rehabil. Med..

[14] Lejczak A (2016). Nordic walking may safely increase the intensity of exercise training in healthy subjects and in patients with chronic heart failure. Adv. Clin. Exp. Med..

[15] Bulińska K (2016). Nordic pole walking improves walking capacity in patients with intermittent claudication: A randomized controlled trial. Disabil. Rehabil..

[16] Dziubek W (2020). Effects of physical rehabilitation on spatiotemporal gait parameters and ground reaction forces of patients with intermittent claudication. J. Clin. Med..

[17] Pietraszewski B, Woźniewski M, Jasiński R, Struzik A, Szuba A (2019). Changes in gait variables in patients with intermittent claudication. Biomed. Res. Int..

[18] Panizzolo FA (2014). Gait analysis in chronic heart failure: The calf as a locus of impaired walking capacity. J. Biomech..

[19] Panizzolo FA (2015). Is the soleus a sentinel muscle for impaired aerobic capacity in heart failure?. Med. Sci. Sports Exerc..

[20] Ozcan EB (2022). Impaired balance and gait characteristics in patients with chronic heart failure. Heart Lung Circ..

[21] Szpala A (2023). Do mechatronic poles change the gait technique of nordic walking in patients with ischemic heart disease?. Appl. Bionics Biomech..

[22] Szpala A (2022). Selected spatiotemporal and joint angle parameters in normal gait and nordic walking with classical and mechatronic poles in aspects of sex differences. Biomed. Res. Int..

[23] Lee R, Akhundov R, James C, Edwards S, Snodgrass SJ (2023). Variations in concurrent validity of two independent inertial measurement units compared to gold standard for upper body posture during computerised device use. Sensors.

[24] Farahan SB, Machado JJM, de Almeida FG, Tavares JMRS (2022). 9-DOF IMU-based attitude and heading estimation using an extended kalman filter with bias consideration. Sensors.

[25] Al Borno M (2022). OpenSense: An open-source toolbox for inertial-measurement-unit-based measurement of lower extremity kinematics over long durations. J. Neuroeng. Rehabil..

[26] Park S, Yoon S (2021). Validity evaluation of an inertial measurement unit (IMU) in gait analysis using statistical parametric mapping (SPM). Sensors.

[27] Bartoszek A, Struzik A, Jaroszczuk S, Woźniewski M, Pietraszewski B (2022). Comparison of the optoelectronic BTS Smart system and IMU-based MyoMotion system for the assessment of gait variables. Acta Bioeng. Biomech..

[28] Szrek, J. *et al.* Force measurement module for mechatronic Nordic walking poles. In *Proceedings of the 14th International Scientific Conference: Computer Aided Engineering. CAE 2018. Lecture Notes in Mechanical Engineering.* (eds. Rusiński, E. & Pietrusiak, D.) 790–794 (Springer, 2019) 10.1007/978-3-030-04975-1_91.

[29] Szpala A (2023). No influence of mechatronic poles on the movement pattern of professional Nordic walkers. Int. J. Environ. Res. Public Health.

[30] Wudarczyk S, Bałchanowski J, Gronowicz A, Szrek J (2020). Research on the mechatronic gait monitoring system with nordic walking poles. Modern Trends in Theory of Machines and Mechatronic Systems.

[31] Wudarczyk S, Woźniewski M, Szpala A, Winiarski S, Bałchanowski J (2023). Mechatronic pole system for monitoring the correctness of Nordic walking. Sensors.

[32] Kaminsky LA, Whaley MH (1998). Evaluation of a new standardized ramp protocol: The BSU/Bruce ramp protocol. J. Cardiopulm. Rehabil..

[33] Hamilton DM, Haennel RG (2000). Validity and reliability of the 6-minute walk test in a cardiac rehabilitation population. J. Cardiopulm. Rehabil..

[34] Uszko-Lencer NHMK (2017). Reliability, construct validity and determinants of 6-minute walk test performance in patients with chronic heart failure. Int. J. Cardiol..

[35] Sharma R, Anker SD (2001). The 6-minute walk test and prognosis in chronic heart failure—The available evidence. Eur. Heart J..

[36] Fletcher GF (2001). Exercise standards for testing and training: A statement for healthcare professionals from the American Heart Association. Circulation.

[37] Shea MG (2022). Comparison of ratings of perceived exertion and target heart rate based exercise prescription in cardiac rehabilitation: Randomized controlled pilot study. J. Cardiopulm. Rehabil. Prev..

[38] Richardson JTE (2011). Eta squared and partial eta squared as measures of effect size in educational research. Educ. Res. Rev..

[39] Cohen, J. *Statistical Power Analysis for the Behavioural Sciences*. *Statistical Power Anaylsis for the Behavioral Sciences* (1988)

